# Exit-Site Care in Pediatric Peritoneal Dialysis Patients: From Personal Preference to Clinical Evidence

**DOI:** 10.1016/j.ekir.2025.09.036

**Published:** 2025-10-01

**Authors:** Bora Gülhan, Ali Düzova

**Affiliations:** 1 Division of Pediatric Nephrology, Department of Pediatrics, Faculty of Medicine, Hacettepe University, Ankara, Türkiye

See Clinical Research on Page 3884

Exit-site infection (ESI) is the leading complication of peritoneal dialysis (PD) and therefore, constitutes the weak link in the chain. If left untreated, ESI may lead to tunnel infection (TI), peritonitis, and even catheter loss. Approximately 20% of all peritonitis cases are preceded by ESI or TI and 15% of ESIs lead to catheter loss.^1^ Colonizing skin flora, including *Staphylococcus aureus* and coagulase-negative staphylococci, account for most pathogens responsible for ESI. Other gram-positive organisms such as *Corynebacterium*, enterococcal species have been associated with ESI. Besides, *Pseudomonas aeruginosa* and other gram negative organisms are responsible for 10% to 30% of ESIs.^1^

For ESIs, a number of treatment and prophylaxis strategies were defined. After catheter implantation, the exit site is kept dry, covered with a sterile dressing, and cleaned with antibacterial soap and/or antiseptic agents. Normal saline, povidone iodine, chlorhexidine, and polyhexamethylene biguanide are used as antiseptic agents. There are mostly adult studies that compare the efficiency of different antiseptic agents.^2^

Beyond antiseptic agents, mupirocin is most commonly used as a prophylaxis agent for gram-positive organisms, especially *S aureus.* It binds to isoleucyl t-RNA synthetase and inhibits bacterial protein synthesis. The routine application of mupirocin, either as part of exit-site care or through repeated intranasal administration, has been inconsistently associated with reductions in *S aureus* nasal carriage and the incidence of *S aureus*–related ESIs and peritonitis. However, mupirocin has little or no effect on *Pseudomonas* or other gram-negative organisms. Gentamicin is another topical agent used for the prevention of ESI.^3^ Obata *et al.*^4^ analyzed 3 randomized control trials comparing mupirocin and gentamicin prophylaxis and reported no significant difference in ESI rates.

Most of the studies support the use of routine PD catheter exit-site care with a sterile cleansing solution and topical antibiotic. However, most of these studies were based on adult studies and pediatric data are scarce. Therefore, the 2024 Pediatric International Society for Peritoneal Dialysis Guideline “suggested” the use exit-site care with a sterile cleansing solution 2 to 3 times/wk and after water exposure, vigorous exercise or soiling of the dressing. The authors “suggested” that topical antibiotics be applied to the catheter exit site whenever exit-site care is performed. The strength of recommendation is level 2 (“we suggest”) and the quality of the supporting evidence is “D” (very low quality). The authors suggested that future studies should aim to better define the balance of risks and benefits associated with different antimicrobial agents, considering both their effectiveness in preventing infection and their potential to promote antibiotic resistance.^5^

In this issue of *KI Reports*, Chan *et al.*^6^ sought to address this gap through a prospective observational cohort study involving pediatric patients (aged < 21 years). This prospective observational study was conducted at their pediatric nephrology center, which is the designated site to provide maintenance kidney replacement therapy for children in Hong Kong. The study was conducted in 2 phases; a preprophylaxis period and a postprophylaxis period, following the introduction of a universal topical antibiotic prophylaxis policy for exit-site care on January 1, 2022. Exit-site cleansing with 0.05% chlorhexidine gluconate solution and dressing changes were carried out under strict aseptic conditions, weekly after the first week and twice weekly during the third and fourth weeks post–catheter placement. Thereafter, all patients performed daily exit-site cleansing with dressing. From January 2022 onward, daily exit-site care included the application of 2% mupirocin ointment. In patients with a history of PD-related infections caused by gram-negative organisms resistant to mupirocin, gentamicin ointment was prescribed according to the susceptibility profile.^6^

They included 57 children; and 39 and 42 patients received PD during the pre- and post-prophylaxis periods, respectively. All patients received automated PD, except 2 children who were on continuous ambulatory PD. As expected, 70% of episodes of ESI/TI were caused by gram-positive organisms. In the preprophylaxis period, 24 episodes of ESI/ TI occurred over 52.4 patient-years. In the postprophylaxis period, 9 episodes of ESI/ TI were reported over 57.7 patient-years (*P* = 0.003). This was a 66% reduction in ESI/TI in the postprophylaxis group. After reduction of gram-positive organisms, the rates of gram-negative, fungal, and culture-negative ESI/TI remained comparable before and after the use exit-site antibiotic prophylaxis.^6^

In the era of evolving antimicrobial-resistant organisms, there has been an interest in the use of medical honey for its antibiotic and wound-healing properties. However, in the literature, there have been different results, Wishart *et al.*^7^ found that exit-site application of medicated honey is more effective than application of povidone iodine to prevent ESI and peritonitis. In contrast, Johnson found no significant difference in ESI or peritonitis rates between topical medicated honey versus intranasal mupirocin.^8^ In the study of Chan et *al.*,^6^ none of the patients had medicated honey; however, the antibiotic susceptibility of *S aureus* to methicillin, gentamicin, and cotrimoxazole remained unchanged. There was more resistance against erythromycin and clindamycin, which were not commonly used antibiotics in the center.^6^

In the study of Chan *et al.*,^6^ there were 1 and 3 peritonitis episodes observed in the pre- and post-prophylaxis periods, respectively; and only 5 catheters were removed in 3 patients. Therefore, it is not possible to draw a certain conclusion about the effect of prophylaxis on catheter survival and peritonitis.^6^

Catheter-related infections are another important cause of hospitalization in patients on PD. Chan *et al.*^6^ reported a 51% reduction in hospitalization days in the postprophylaxis group. The rates of hospitalization episodes were not different between groups. This has contributed to medical cost savings of the preventive prophylaxis group and the estimation was $1342.2 per patient-year. This points out the positive impact of this strategy on health expenditure in pediatric patients on PD.^6^

The recommended target for the overall ESI rate is ≤ 0.4 episodes/patient-yr and this study had higher ESI/TI incidence (0.46 episodes/patient-yr) at the preprophylaxis period.^9^ Despite relatively small sample size and the relatively low percentage of young children (< 2 years, 7%; 2–8 years, 16%) who are at increased risk of infectious complications, Chan *et al.*^6^ provide a prominent prospective study for the effectiveness of antibiotic prophylaxis in pediatric PD patients to reduce ESI/TI rates.

## Disclosure

All the authors declared no competing interests.
